# A Bio-Based Composite Hydrogel Substrate for Indoor Soilless Dandelion Cultivation: Growth Performance and Polysaccharide Accumulation

**DOI:** 10.3390/gels12030235

**Published:** 2026-03-12

**Authors:** Yongxin Guo, Jianxun Ma, Yuhan Zheng, Gang Wang, Hongda Zhang, Yong Yu, Jinpeng Zhang

**Affiliations:** 1 Jilin Provincial Key Laboratory of Environmental Ecology in Black Soils, Research Center of Regional Development and Environment, Northeast Institute of Geography and Agroecology, Chinese Academy of Sciences, Changchun 130102, China; guo15981389011@163.com (Y.G.); zhanghongda@iga.ac.cn (H.Z.); yuyong@iga.ac.cn (Y.Y.); 2 University of Chinese Academy of Sciences, Beijing 100049, China; 3 College of Life Science, Jilin Agricultural University, Changchun 130118, China; 20232135@mails.jlau.edu.cn (J.M.); zhengyuhan1121@163.com (Y.Z.); wanggang@jlau.edu.cn (G.W.)

**Keywords:** hydrogel, Kelcogel, pectin, dandelion, soilless cultivation

## Abstract

Sustainable agricultural techniques can ensure food security around the world. Hydrogel based soilless culture is an ecological and efficient alternative compared to conventional agriculture. Here, a multi-component hydrogel (pectin, Kelcogel, and chitosan/Se hydrogel, PKCH) was prepared by synthesizing natural biomolecules to cultivate dandelion for stimulate dandelion growth and improve nutritional value. The germination percentage of dandelion on PKCH (88.89%), was significantly higher than that in traditional hydroponics and pure Kelcogel (*p* < 0.05). Compared with hydroponics, the long-term dandelion cultivation experiments demonstrated that the PKCH cultivation mode enhanced root vitality, further increasing the growth and yield of dandelions (shoot length: 18.36 ± 0.30 cm, root length: 9.28 ± 0.21 cm, main root diameter: 0.94 ± 0.02 cm). The hydrogel substrate was associated with improved nutrient solubilization and sustained release, which may be linked to the accumulation of low-molecular-weight organic acids in the rhizosphere. Exogenous Se was effectively assimilated and transported to the above-ground parts of dandelion, which stimulated the photosynthetic efficiency and nutritional accumulation of dandelion. The polysaccharide content of dandelion reached 69.40 ± 0.13% (expressed as glucose-equivalent total sugars), which demonstrated the potential antioxidant properties and medicinal value. Technical economic analysis revealed the cost-effectiveness of PKCH synthesis and application. This study enriches the application of hydrogels in dandelion cultivation and provides an alternative approach for cultivating dandelion in soilless environments and medicinal crop production techniques.

## 1. Introduction

Soilless cultivation technique, as an important development direction of modern agriculture, has demonstrated significant advantages in increasing crop yields and achieving precise environmental control [1]. The hydroponic systems have become the mainstream technique due to the environmental control and compatibility [2]. However, current hydroponic propagation systems still face key challenges such as high infrastructure costs (with cultivation tanks and pumps accounting for >30% of operational expenses), complex nutrient management, and unstable synthesis of secondary metabolites, which severely restrict their application in the high-quality production of medicinal plants [3,4,5,6]. Hydrogels are an effective solution to problems, including the limitations of traditional cultivation systems, due to the unique molecular network structure and excellent water retention performance [7,8]. Notably, multi-component hydrogel systems can effectively address the stability deficiencies of single-component systems through functional synergy. Specifically, for high-value medicinal crops like dandelion, hydrogel substrates provide a solution to the stringent hydration and nutrition demands while optimizing bioactive compound production. The unique β-1,3-glucan backbone of dandelion exhibits nanofibrillar morphology (20–50 nm), enhancing bioavailability and bioactivities such as antioxidant and anti-inflammatory properties [9,10]. Critically, the metabolic modulation of dandelion crude polysaccharides under hydrogel-mediated cultivation remains unexplored—a key research gap addressed in this study. Conventional soil cultivation limits yield consistency and bioactive potency, whereas hydrogel-based systems reduce resource dependence while minimizing nutrient runoff.

Additionally, natural hydrogels are particularly advantageous for sustainable agriculture due to their biodegradability and cost-effectiveness [11,12,13,14]. The commonly utilized hydrogels at present include cellulose [15,16], chitosan [17], alginate [18], pectin [19,20], and phytagel [21]. Among various natural polymers, pectin has attracted much attention due to its excellent hydrophilicity and biocompatibility, which are endowed by its rich oxygen-containing functional groups [22]. Compared with materials such as alginate, pectin offers better cost-effectiveness [23] and shows potential for sustainable agricultural applications. However, the gel-forming process must be conducted under acidic conditions, which imposes certain constraints on crop growth [24]. Low acyl Phytagel (Kelcogel™) is an anionic linear polysaccharide produced by microbial fermentation. Its fundamental structure consists of four repeating monosaccharide units: two glucose molecules, one glucuronic acid, and one rhamnose, with a largely unbranched backbone. The production of Kelcogel entails the deacylation of natural phytagel via alkali treatment. The gel formation of phytogels can be accomplished by adding MgCl_2_ or CaCl_2_ [25]. Moreover, crop growth could be promoted by Se-doped chitosan substrates due to the enhanced antioxidant capacity and stress resistance. This multi-component system achieves multiple objectives, including intelligent water management, precise selenium nutrient supply, soil ecological regulation, and plant immune activation.

In this study, a ternary composite hydrogel system was innovatively developed by combining pectin, Kelcogel, and Se/chitosan, which was incorporated into the hydrogel substrate (pectin-gel-chitosan hydrogel, PKCH) to augment the nutritional profile of dandelion. This study aims to evaluate the performance of PKCH hydrogels in enhancing dandelion growth compared to conventional hydroponics, with a focus on water retention, nutrient delivery, and mechanical strength. The nutritional value of dandelion was assessed by measuring polysaccharide content. The chemical interaction between crop roots and PKCH was studied by analyzing root exudates (oxalic acid and acetic acid). Technical economic analysis (TEA) and life cycle assessment (LCA) were used to investigate the cost-effectiveness and environmental impact of the PKCH system. This work evaluates the application of PKCH in enhancing the growth and medicinal value of dandelion and provides useful data for soilless cultivation and medicinal crop production techniques.

## 2. Results and Discussion

### 2.1. Preparation and Characterization of PKH and PKCH

**Figure 1:**
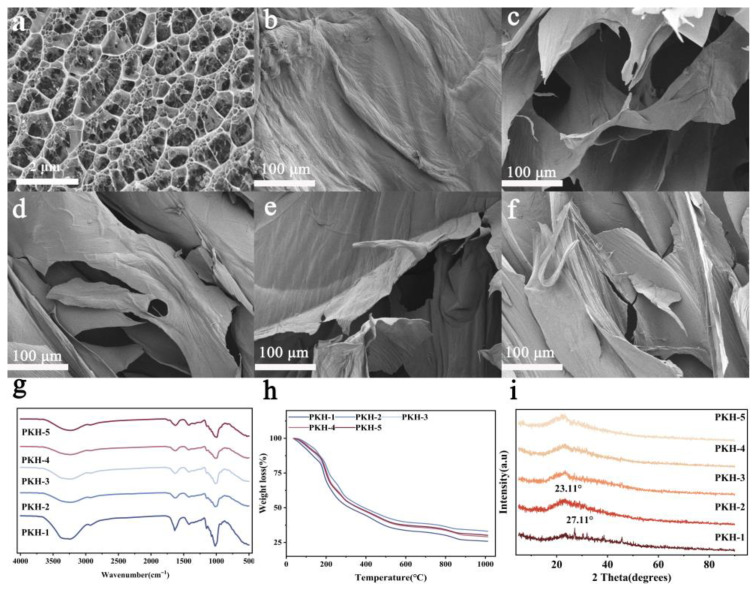
SEM images of prepared hydrogels: Kelcogel (**a**), PKH-1 (**b**), PKH-2 (**c**), PKH-3 (**d**), PKH-4 (**e**), PKH-5 (**f**), FT-IR (**g**), TGA (**h**), XRD (**i**) curves of prepared PKHs.

The prepared PKH (pectin/Kelcogel hydrogel) was observed by SEM. The PKH was examined after the addition of different concentrations of pectin. The surface of pure Kelcogel (PKH-1) was smooth and free of pores and cracks (Figure 1). Kelcogel presented a uniform porous structure (Figure 1a). After the addition of pectin, the surface of the PKH composite hydrogel showed cracks and a porous structure, indicating that pectin was effectively incorporated into the hydrogel network and exhibited a uniform distribution on the hydrogel surface (Figure 1b–f). With the increase in pectin dosage, more voids were formed, which was advantageous for the transport of water and nutrients. Root growth was promoted within PKH. The hydrogel, with its porous structure, is essential for soilless culture applications.

Molecular interactions (PKHs) were studied by FT-IR (Figure 1g). The results showed that the peaks at 2926 cm^−1^ and 1746 cm^−1^ were attributed to C-H and C=O stretching vibrations, respectively, while the broad characteristic band at 3330 cm^−1^ corresponded to the O-H stretching vibration of pectin in Kelcogel-based hydrogels. The peak at 1146 cm^−1^ was assigned to the C-O stretching of COOH groups. The absorption band at 1060 cm^−1^ was associated with the stretching vibration of the α-(1,4)-glycosidic bond, indicating that the interaction between the pectin network and the Kelcogel lattice occurred via hydrogen bonding and van der Waals forces without altering the functional groups of pectin and Kelcogel, and confirming good compatibility and strong binding between pectin and Kelcogel.

The thermal stability of PKH hydrogels was analyzed by TGA (Figure 1h). Thermal decomposition of Kelcogel and PKH was examined (Table A1). Stage 1 involved thermal degradation of the samples, resulting in a 16% weight loss between 30 °C and 173 °C. Stage 2 resulted in a 55% weight loss. The first stage of weight loss occurred between 30 °C and 173 °C due to water evaporation. The thermal decomposition of PKH might be associated with the second stage of degradation, which occurred between 173 °C and 1000 °C and led to a weight loss of 55%. At 1000 °C, PKH still retained approximately 30% residual weight, indicating excellent thermal stability.

The structures of Kelcogel and PKH were characterized by XRD, which exhibited a peak at 27.11°, reflecting the amorphous characteristic of Kelcogel (Figure 1i). The PKH showed a broad peak at 23.31°, and no other peaks were observed. Thus, the crystallinity of PKH remained unchanged even when pectin was incorporated into the polymer. The diffraction peaks of PKH fell within the same range with a slight shift, indicating that the atomic arrangement and lattice spacing were basically similar for all pectin-derived samples.

The FT-IR spectra of chitosan and chitosan/Se (Figure A2) show that the absorption peaks of 3450 cm^−1^ and 2917 cm^−1^ were attributed to the O-H stretching vibrations and C-H stretching vibrations. The absorption peaks of 1653 cm^−1^ and 1598 cm^−1^ were attributed to N-H bending vibration and C=O stretching vibration. An absorption peak was shown at 807 cm^−1^ in Chitosan/Se. This phenomenon could be due to the telescopic vibration of Se=O, indicating that the reaction formed a selenite bond. Meanwhile, the disappearance of the C_6_-OH peak at 1025 cm^−1^ and the unchanged C_3_-OH peak at 1157 cm^−1^ were attributed to the selenation reaction of C_6_-OH. Additionally, the C-N and N-H peaks were shifted by the protonation of N-H in chitosan and the adsorption of Se in solution. The results showed that Se was introduced into the chitosan skeleton by electrostatic attraction of the amino groups of chitosan and esterification of C_6_-OH.

The BET surface area of PKH hydrogels was illustrated in Figure A3, and the corresponding specific surface areas and total pore volumes were presented in Table A2. The N_2_ adsorption isotherms of PKH exhibited a rapid increase in adsorption at low P/P_0_, followed by a plateau at intermediate P/P_0_, which is characteristic of type-IV isotherms. These findings suggest that the incorporation of pectin into PKH enhanced the specific surface area and pore structure of the composite hydrogel. The specific surface area of PKH increased from 28.10 m^2^/g to 47.29 m^2^/g. Furthermore, the addition of pectin increased the pore volume of the composite hydrogel from 0.054 cm^3^/g to 0.093 cm^3^/g. The results corroborate that PKH-5 possesses a considerable surface area and porosity. The addition of pectin introduced additional –COOH groups into the PKCH hydrogel, which were cross-linked with Kelcogel via Ca^2+^-mediated bonding, further enhancing the structural integrity of the entire network. The incorporation of pectin chains disrupted the tight packing of Kelcogel during gel formation, creating interstitial pores and interconnected channels, thereby increasing the overall porosity. The high specific surface area facilitates contact with and adsorption of more water molecules, thereby promoting the transport of water and nutrients within the PKH matrix. This enhanced hydration stimulated seed water uptake, further promoting plant growth. The mesoporous scaffolds facilitated water distribution and nutrient release. Additionally, their high specific surface area provided abundant active sites for the adsorption and ion exchange of nutrients, including controlled release of Se. These physical and chemical properties may promote the development of the root elongation zone and reduce growth variability among seedlings.

**Figure 2:**
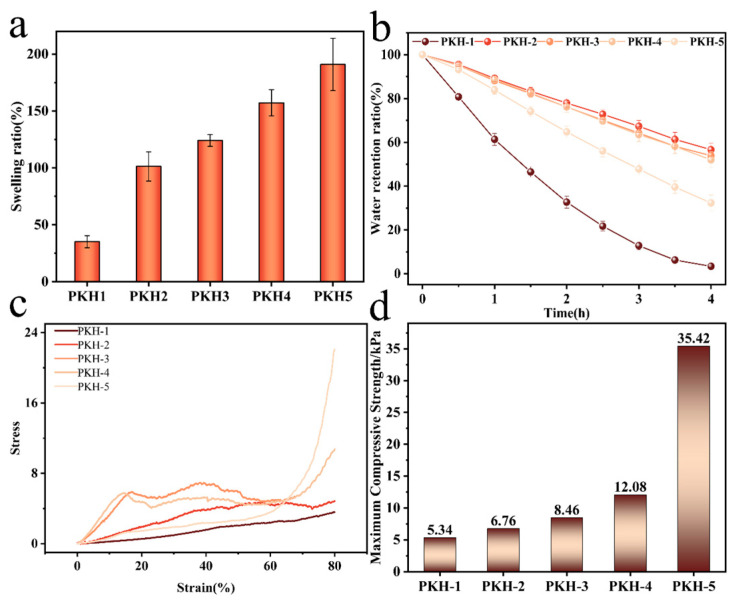
Swelling ratio (**a**), deswelling ratio (**b**), compressive stress–strain curve (**c**), and maximum compressive strength (**d**) of PKH 1-5.

The results showed that the pectin content was positively correlated with the swelling rate of PKH. The swelling ratios of PKH-1 to PKH-5 after 12 h were 35.16 ± 5.31%, 101.31 ± 12.81%, 124.17 ± 5.19%, 157.18 ± 11.52%, and 190.87 ± 22.93%, respectively. The increase in pectin concentration resulted in higher water absorption capacity due to improved cross-linking of the polymer network (Figure 2a). In soilless culture, water uptake capacity from the environment was critical for growth. The presence of hydroxyl groups in pectin made the PKH more hydrophilic, thereby enhancing its water uptake capacity. The results suggested that pectin improved water uptake capacity. The water retention of PKH-5 was higher than that of PKH-2-4 (Figure 2b). Therefore, the utilization of PKH-5 as a substrate for soilless culture was the most appropriate choice. The appropriate amount of pectin could enhance the stability of the substrate, collect water more efficiently, and ensure moisture for the plant root system.

The stress–strain curves of the prepared hydrogels showed that the fracture stress increased with increasing pectin concentration (Figure 2c,d). Pectin concentration was positively correlated with the maximum compressive stress of the hydrogels, which was 5.34, 6.76, 8.46, 12.08, and 35.42 kPa for PKH-1 to PKH-5, respectively. The highest compressive value was observed for PKH-5. The addition of pectin improved the stress transfer characteristics of the matrix. In addition, pectin-induced resistance helped reduce the deformability of the Kelcogel macromolecules, suggesting that the presence of pectin reduced the mobility of the composite hydrogel.

### 2.2. Growth Evaluation of Dandelion

**Figure 3:**
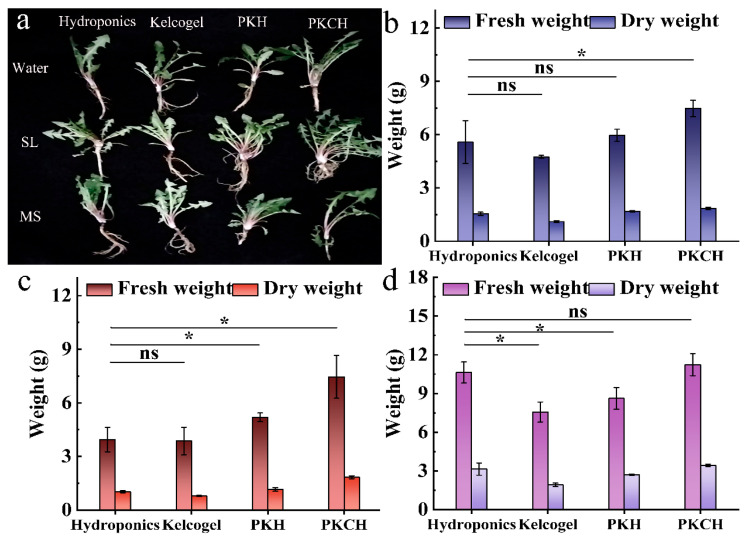
Dandelion growth (**a**), dry weight and fresh weight of dandelions: traditional hydroponics (**b**), soil leachate culture (**c**), MS nutrient solution culture (**d**) (* *p* < 0.05, ns > 0.05).

Growth tests of dandelion included three solution treatments and three substrate treatments. All treatment groups grew well after seed germination (Figure 3a and Table A3). By observing the germination rates in different media over a period of 0–20 days, it was found that the addition of MS solution better promoted seed germination, while increased pectin and chitosan also improved the germination rate. The porous and hydrophobic structural domains on PKH-5 and PKCH surfaces allowed water to penetrate and stimulated plant germination. Oxygen-containing functional groups in PKH and PKCH also facilitated water absorption. The group of PKCH/MS exhibited the highest germination rate, demonstrating that hydrogel-based cultivation could limit competitive nutrient uptake among plants compared to hydroponic cultivation, thereby enhancing seed germination. Furthermore, the addition of Se also favored dandelion growth (Figure A4).

The effects of different fractions of substrates on plant fresh and dry weights were investigated (Figure 3a–d and Table A4). The SL treatment group (Table A5) showed lower moisture content than other treatment groups (*p* < 0.05), which could be attributed to the alkaline nature of the soil samples, making them unfavorable for dandelion cultivation. The variation in fresh and dry weight results indicated that the differences in root and stem length between the DI water group and hydrogel cultivation did not affect water uptake in dandelions.

**Figure 4:**
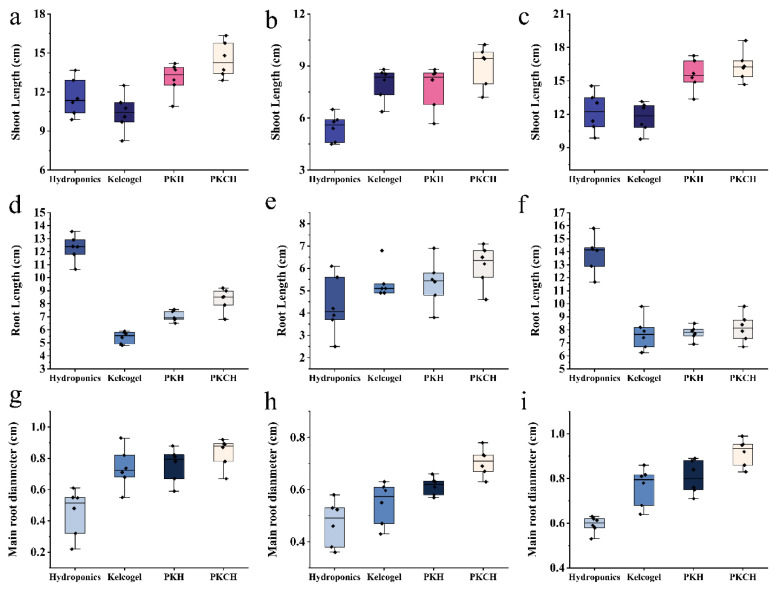
The changes in shoot length of seedlings: traditional hydroponics (**a**), soil leachate culture (**b**), MS nutrient solution culture (**c**); root length of seedlings: traditional hydroponics (**d**), soil leachate culture (**e**), MS nutrient solution culture (**f**); diameter of the main root of seedlings: traditional hydroponics (**g**), soil leachate culture (**h**), MS nutrient solution culture (**i**).

The stem length of dandelion grown in the PKCH group (8.53 ± 0.44 cm and 13.7± 0.81 cm) was shorter than that of the control group (12.36 ± 0.44 cm and 11.51 ± 1.04 cm), indicating that in the control group, the roots and stems of the seedlings were not limited by water transfer (Figure 4a–c and Table A6). However, in the case of hydrogel cultivation, the root system of dandelion seedlings was shorter than that of the DI water group due to competition for water between the hydrogel and the plant root system, and the mechanical strength of the gel hindered the growth of the elongation zone of the root system (Figure 4d–f). The diameter of the elongation zone of the primary root was much larger than that of the control group seedlings (Figure 4g–i).

Compared to the DI water group, hydrogel cultivation limited the transfer and competition for plant nutrients in the substrate, thereby increasing the efficiency of plant nutrient uptake by seedlings and reducing growth differences among seedlings within a group. The majority of PKH and PKCH substrates were composed of crude polysaccharides. The PKH and PKCH could carry chemicals and control their diffusion process inside the system. The structure of PKH and PKCH created a physical barrier for nutrients. In addition, root and stem lengths were close to those of the DI water group, confirming the great cultivation potential of PKCH composite hydrogels.

In the PKCH-treated group in MS, root and stem length, and diameter of the primary roots (16.16 ± 0.57 cm, 8.71 ± 0.21 cm, and 0.95 ± 0.02 cm) were significantly higher than those in the PKH-5-treated group (15.66 ± 0.81 cm, 8.03 ± 0.33 cm, and 0.84 ± 0.06 cm) (*p* < 0.05), indicating that chitosan/Se could promote the development of dandelion seedlings and improve their nutritional value (Figure 4i). The mechanical properties of PKH and the addition of chitosan/Se resulted in a more complex network structure. Results showed that pectin optimized the mechanical properties and structure of the hydrogel, which had a positive effect on the planting of dandelion seedlings. Furthermore, interaction effects between substrate type and nutrient solution composition were observed for multiple growth parameters. These results suggested that the growth-promoting effects of PKCH are amplified when combined with MS, highlighting the importance of optimizing substrate–nutrient solution pairings in soilless cultivation.

### 2.3. Long-Term Cultivation Experiment and Component Analysis

**Figure 5:**
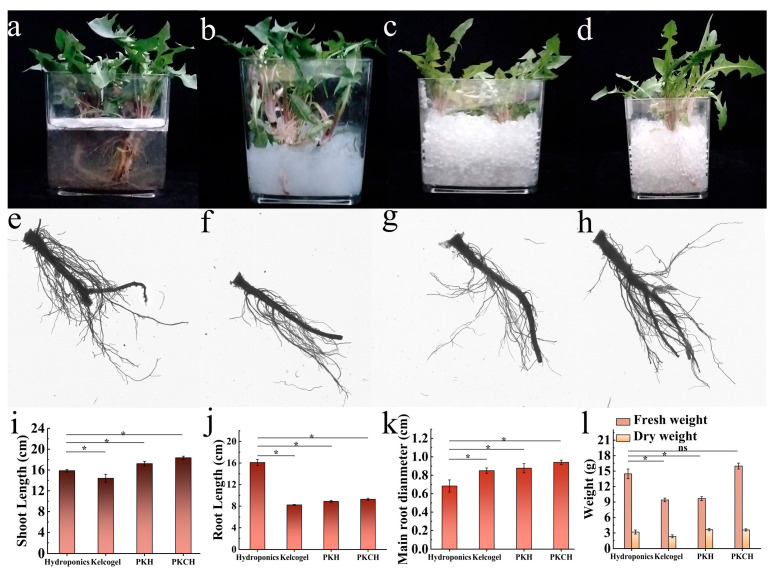
Dandelion cultivation in 45 d: hydroponics (**a**), Kelcogel (**b**), PKH (**c**) and PKCH (**d**); root scan images of dandelion: hydroponics (**e**), Kelcogel (**f**), PKH (**g**) and PKCH (**h**); shoot length (**i**), root length (**j**), diameter of the main root (**k**), dry weight and fresh weight (**l**) of seedlings (* *p* < 0.05, ns > 0.05).

To ascertain the stability of PKCH in soilless culture, the planting period was extended to 45 d. The effect of PKCH was evaluated in long-term cultivation by determining root and stem length, water content, and root diameter over a period of 45 d (Figure 5a–d). In the long-term dandelion cultivation experiment, PKCH resulted in stem length, root length, and main root diameter of 18.36 ± 0.30 cm, 9.28 ± 0.19 cm, and 0.94 ± 0.02 cm, respectively (Figure 5e–h), further confirming the feasibility of Kelcogel, PKH, and PKCH for long-term application in dandelion cultivation (Figure 5i–l and Table A7 and Table A8). Replicated experiments revealed that PKCH has reuse value in dandelion cultivation and provides significant economic benefits. In comparison, the recirculating nutrient film technique is still the mainstay of dandelion soilless cultivation in Bioregenerative Life Support Systems [26]. This technique requires complex ventilation and nutrient delivery systems. The PKCH hydrogel cultivation technique avoids the need for complex equipment and optimizes the cultivation process.

**Figure 6:**
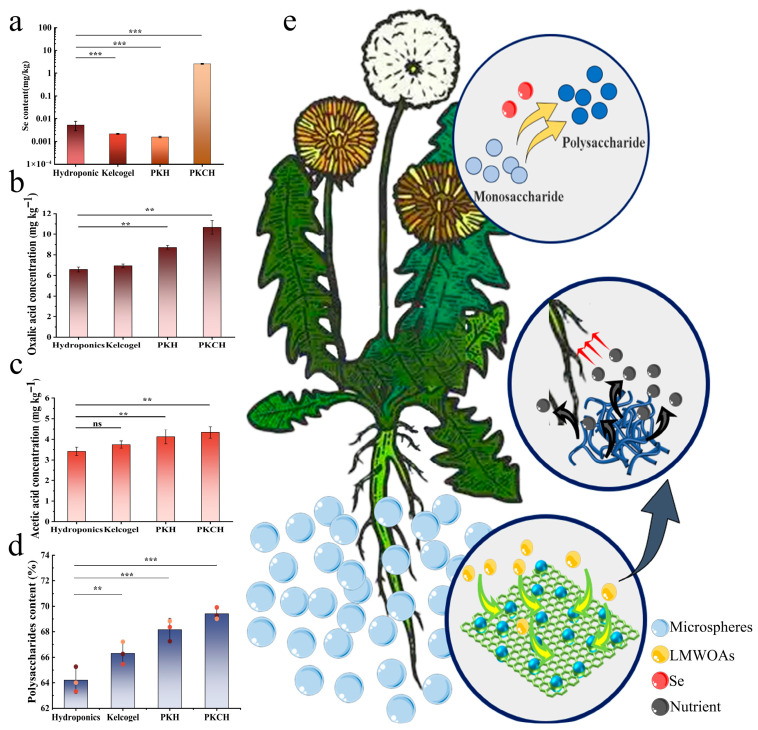
Se content (**a**); oxalic acid concentration (**b**); acetic acid concentration (**c**); polysaccharide content (**d**); the promotion of the growth and development of dandelion and the improvement of nutritional value by PKCH (**e**) (*** *p* < 0.001, ** *p* < 0.01, ns > 0.05).

Exogenous Se was efficiently absorbed by seedlings and translocated into plant tissues, significantly enhancing aboveground biomass (*p* < 0.05) (Figure 6a). The combined treatment of pectin and Kelcogel activated organic acid metabolic pathways, inducing specific alterations in the composition of low-molecular-weight organic acids (LMWOAs) in dandelion root exudates (Figure 6b–c and Table A9). Both the PKCH and PKH groups exhibited markedly higher oxalate and acetate concentrations compared to the control group (*p* < 0.01). The accumulation of LMWOAs might increase the pectin methylesterification degree and reduce Ca^2+^-pectin network cross-linking strength through chelation [27]. Consequently, the hydrogel substrate exhibited improved nutrient solubilization and sustained release, optimizing mineral bioavailability for dandelion uptake. Under the pectin-Kelcogel-Se tripartite synergy, crude polysaccharide content in the PKCH group showed statistically significant differences (*p* < 0.05) relative to both the PKH group and the control, suggesting that this system optimizes plant nutrient uptake efficiency through dynamic regulation of carbon allocation strategies (Figure 6d). The incorporation of Se into a PKCH system demonstrated a pronounced impact on dandelion growth and polysaccharide biosynthesis [28,29].

**Figure 7:**
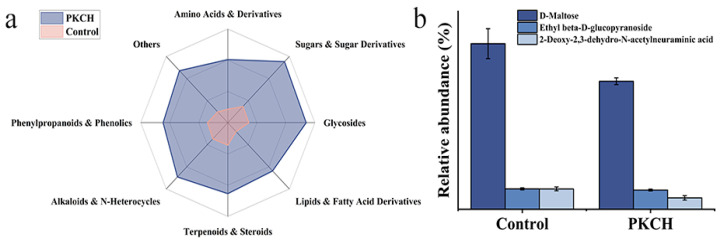
Relative abundance differences in total small molecule metabolites (**a**); Relative abundance differences in sugars & sugar derivatives (**b**).

The small-molecule metabolites of the whole dandelion plant were detected by LC-MS to investigate metabolite variations under different cultivation regimes. Results demonstrated that the application of PKCH activated biological metabolic pathways in dandelions, leading to an increase in the relative abundance of small-molecule metabolites (Figure 7a and Figure A5). Elevated levels of D-maltose, ethyl β-D-glucopyranoside, and 2-deoxy-2,3-dehydro-N-acetylneuraminic acid further supported the stimulatory effect of PKCH on polysaccharide metabolism in dandelions (Figure 7b). These three metabolites corresponded respectively to substrate supply (D-maltose), key enzyme activity (ethyl β-D-glucopyranoside), and specialized modification pathways (neuraminic acid derivatives). The accumulation of D-maltose indicated an increase in carbon source availability for dandelion polysaccharide synthesis, suggesting that the synthetic capacity of glucans—such as starch and inulin (the primary storage polysaccharides in dandelions)—was enhanced under the PKCH culture mode [30]. The elevated levels of ethyl β-D-glucopyranoside indicated an enhanced ability of dandelion cells to construct complexes such as polysaccharide–protein and polysaccharide–polyphenol conjugates. The PKCH cultivation system systematically enhanced the nutritional value potential of dandelions by promoting the accumulation of these three characteristic metabolites.

### 2.4. Technical Economic Analysis and Life Cycle Assessment

**Figure 8:**
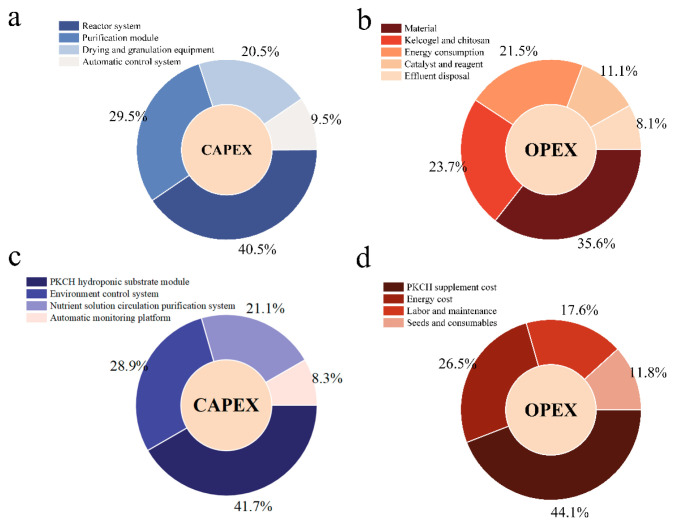
TEA of synthesis system of PKCH: CAPEX (**a**) and OPEX (**b**); soilless cultivation system of PKCH: CAPEX (**c**) and OPEX (**d**).

To evaluate the feasibility of soilless cultivation of dandelion using PKCH for large-scale application, the synthesis system and soilless cultivation system of PKCH were analyzed through the ASPEN PLUS V11 model with TEA and LCA (Table A10, Table A11, Table A12 and Table A13). The boundary of the PKCH synthesis system was defined as gate-to-gate, whereas the hydroponic cultivation system was assessed on a gate-to-grave basis. The LCA adopted a cradle-to-grave perspective, covering the entire life cycle of the system. Key assumptions in the Aspen Plus V11 model (including feedstock composition, reactor yield, and thermodynamic methods) were based on experimental data and standard databases. The energy structure assumption used in the LCA was derived from regional grid data, which formed the basis for calculating the 38% reduction in life cycle greenhouse gas emissions. To verify the robustness of the conclusions, we conducted a sensitivity analysis, systematically examining the impact of key parameters such as raw material costs, drying energy consumption, and energy structure on the results. The reactor system in the PKCH synthesis system accounts for 40.48% of the capital expenditure (CAPEX) based on TEA (Figure 8a). The raw material cost of PKCH accounts for 44.12% of the operating expense (OPEX) (Figure 8b). In the soilless cultivation system, the hydroponic substrate module accounts for 41.67% of the CAPEX, primarily involving materials and controlled-release design (Figure 8c). The supplementary cost of PKCH accounts for 44.12% of the total OPEX. The economic viability (high-value-added product) and sustainability (38% reduction in lifecycle emissions) of PKCH were confirmed by LCA (Figure 8d). The drying process remains the main bottleneck in energy consumption, necessitating exploration of low-temperature drying or waste heat recovery techniques.

## 3. Conclusions

Low-cost natural plant polysaccharide (pectin) and microbial polysaccharide (Kelcogel) were synthesized into PKCH via a cross-linking method. PKCH exhibited a three-dimensional lattice-like structure, excellent thermal stability, and hydrophilic properties. The addition of pectin greatly improved the mechanical strength of the material. The abundant oxygen-donating functional groups and the stability in multicomponent hydrogels promoted seed germination and plant growth. Compared with the hydroponic system, the PKCH system further stimulated dandelion growth (shoot length: 18.36 ± 0.30 cm, root length: 9.28 ± 0.21 cm, main root diameter: 0.94 ± 0.02 cm) and polysaccharide metabolism (69.40 ± 0.13%). The preparation of PKCH overcame the limitations of single-component hydrogels, and the synergistic stimulation strategy of pectin-Kelcogel-Se effectively promoted the metabolism of LMWOAs and the synthesis of crude polysaccharides in dandelion. This study was conducted under controlled conditions, and the performance of the hydrogel in real agricultural environments (e.g., outdoor or large-scale systems) remains to be investigated. The long-term durability and performance of hydrogels need to be explored, as well as their effectiveness on other crops or species. Furthermore, the mechanical strength of the hydrogel depends on the nature of its material. The use of natural, low-stability hydrogels is expected to optimize root elongation. This study has significant implications for the development of cost-effective, eco-friendly substrates for large-scale hydroponic systems, potentially reducing dependency on traditional soil-based agriculture and contributing to more sustainable farming practices.

## 4. Materials and Methods

### 4.1. Materials

Kelcogel was acquired from Thousand Flavors Foods Corporation, Guangzhou, China. Pectin (from citrus peel, ~210,000 Da, methoxy groups ≥ 6.7%, galacturonic acid ≥ 74.0%, degree of esterification: 20–50%) was acquired from Sigma, Shanghai, China. Chitosan (~100,000 Da, degree of deacetylation: 90.389%) was acquired from Aladdin, Shanghai, China. All hydrogels used were employed for hydrogel preparation without further purification. Dandelion seeds were obtained from the Chinese Academy of Agricultural Sciences. MgCl_2_·6H_2_O, Na_2_SeO_3_, acetic acid, and Murashige and Skoog culture medium (MS) were analytical grade (AR) and purchased from Aladdin, Shanghai, China. Deionized water (DI water) was used in the laboratory.

### 4.2. Preparation of Hydrogels

Preparation of Chitosan/Se: 0.5 g of chitosan was dissolved in DI water (40 mL), and glacial acetic acid (1 mL) was added to the solution and stirred. The Na_2_SeO_3_ (0.536 g) was dissolved in 10 mL of DI water. The chitosan solution was slowly added to the sodium selenite solution and stirred at 70 °C for 6 h. The resulting chitosan/Se product was dialyzed against water, followed by washing with 75% ethanol and lyophilization.

Preparation of PKH1-5: Pectin/Kelcogel hydrogels at different concentrations (0:1, 1:4, 1:3, 1:2, 1:1, wt%) were prepared for PKH-1 to PKH-5. For example, PKH-5 was prepared by thoroughly mixing pectin (1.2 wt%) and Kelcogel (1.2 wt%) in DI water (1 L). Moreover, PKCH was prepared by adding chitosan/Se (0.02 wt%) into the PKH solution and stirring at 95 °C for 1 h. Then, 200 mL of MgCl_2_ (10 mM) was added to the gel solution. The PKHs were poured into polytetrafluoroethylene molds and cooled at 25 °C to induce coalescence of the gel network.

### 4.3. Characterization of Hydrogels

Functional group characterization of pectin, Kelcogel, and PKCH was analyzed using an FT-IR spectrometer (Thermo Scientific^TM^, NICOLET IS5, Waltham, MA, USA) with a wavelength range from 400 to 4000 cm^−1^ and the potassium bromide disk (thin pellet) technique. The XRD measurements were performed at 40 kV and 30 mA over a 2θ range of 10–80° (D8 ADVANCE, Bonney Lake, WA, USA, Bruker) by Cu Kα radiation (λ = 1.5406 Å) as the X-ray source. Weight loss of hydrogels with different compositions was evaluated under a N_2_ atmosphere from 30 °C to 1000 °C at a heating rate of 10 °C/min by Thermogravimetric Analysis (DISCOVERY SDT 650, TA Instruments, New Castle, DE, USA). All the samples were subjected to freeze-drying treatment and then gold-coated to enhance conductivity. The prepared hydrogels were characterized by SEM (GEMINI 300, Carl Zeiss AG, Oberkochen, Germany). The Brunauer–Emmett–Teller (BET) surface area and Barrett–Joyner–Halenda (BJH) pore size distribution of the samples were determined at 273 K using Quadrasorb Evo^TM^, Quantachrome, Boynton Beach, FL, USA). Prior to analysis, the samples were subjected to freeze-drying treatment and degassed at 65 °C for 5 h to remove adsorbed molecules from the surface and pores.

### 4.4. Swelling, Deswelling and Mechanical Properties

The PKH-1 to PKH-5 samples were soaked in DI water for 12 h at 25 °C to reach equilibrium, and the swelling ratio (SR, %) was then calculated using the following Equation (1):

(1) SR%=Ms−MdMd×100

where M_d_ is the mass of the dried hydrogel and M_s_ is the mass of the swollen hydrogel.

The deswelling behavior of hydrogels with different compositions was tested at 50 °C. The samples, after lyophilization, were immersed for 12 h to achieve equilibrium. The samples were then dried, and the weight change was recorded at specific time intervals. The weight was recorded every 0.5 h. The formula for calculating the deswelling rate (DR) is given in Equation (2):

(2) DR%=WtW0×100

where W_0_ is the weight of the swollen hydrogel at room temperature and W_t_ is the weight of the hydrogel after 0.5 h of placement in the vacuum drying oven.

The compressive strength tests were conducted by a testing machine (CMT6103, MTS System Corporation, Eden Prairie, MN, USA) at 25 °C with a crosshead speed of 10 mm/min. The hydrogels were molded into cylindrical specimens (27 × 20 mm). The mechanical properties of the materials were evaluated based on stress–strain curves.

### 4.5. Growth and Metabolism of Dandelion

The DI water (500 mL) was poured into a transparent container, and the container was covered with gauze. Dandelion seeds were soaked in 20 °C warm water for 6–8 h, and then placed in a 15 °C environment for 24 h to break dormancy.

**Figure A1:**
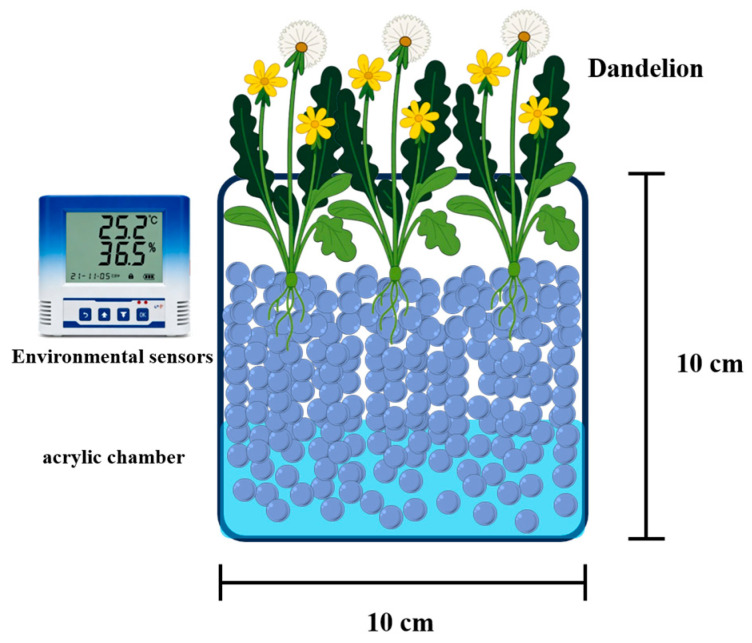
Schematic diagram of the experimental setup.

**Figure A2:**
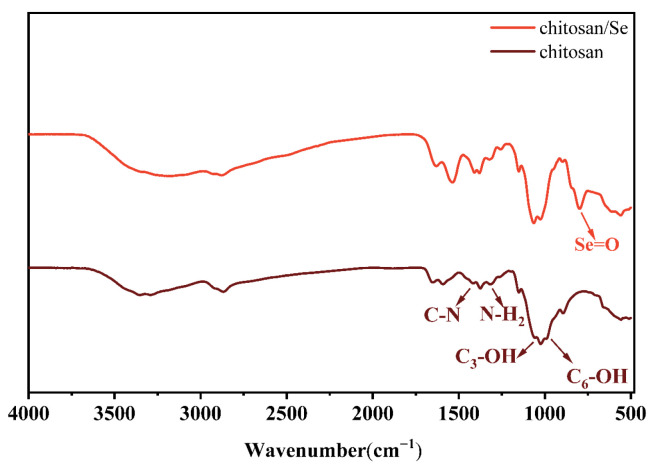
FT-IR spectra of prepared chitosan/Se.

**Figure A3:**
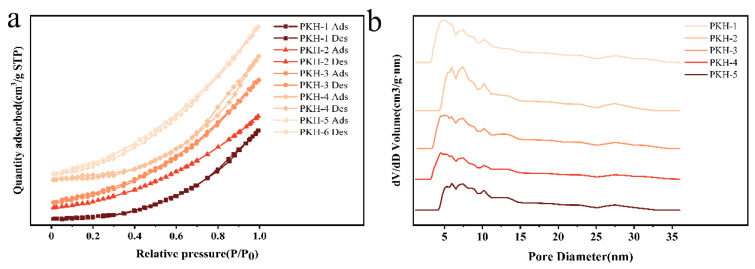
Surface area (**a**) and pore diameter (**b**) of PKH 1-5.

**Figure A4:**
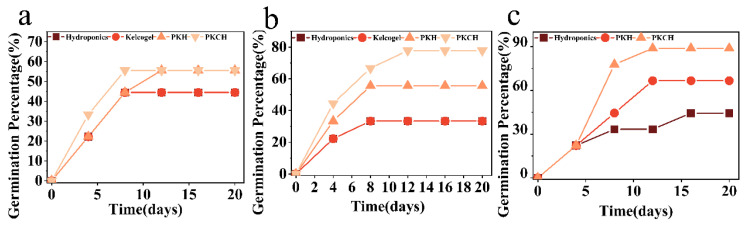
Germination percentages under different treatments for soilless cultivation: DI water (**a**); SL solution (**b**); MS (**c**).

**Figure A5:**
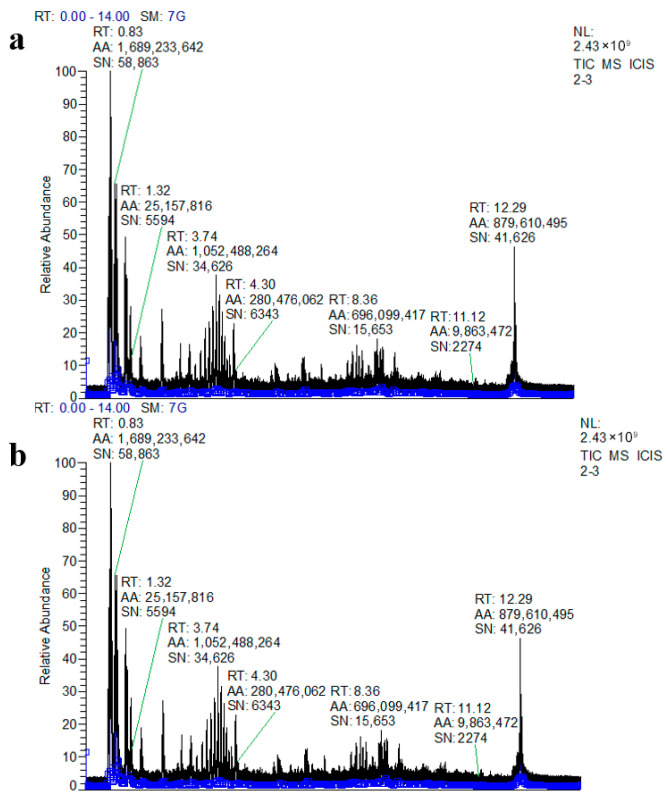
LC-MS of small-molecular metabolites in hydroponic (**a**) and PKCH systems (**b**).

Seed of dandelion were conducted in an acrylic chamber (10 × 10 × 10 cm). To investigate the effects of different substrates on the growth of dandelion, Kelcogel, PKH, and PKCH were employed in a culture system. Furthermore, DI water, Soil Extraction liquid (SL), and MS medium were utilized to explore the impact of nutrient media on dandelion development. Environmental sensors were used to monitor and record the temperature and relative humidity in the culture area (ranging from 15 to 28 °C and 20% to 40%). Key growth indicators were recorded through regular manual measurements. A root length greater than 1 cm was considered evidence of germination. After 20 days of cultivation, plants were collected, and root length, stem length, fresh weight, and dry weight were measured. The lighting system relied entirely on natural sunlight (14.73–22.05 kW m^−2^ d^−1^); no artificial lighting was utilized, and the natural day–night cycle (9–10 h) was followed. The irrigation method employed a one-time bottom-watering system: 250 mL of DI water was added to the bottom of the culture container. The hydrogel substrate absorbed water from below via capillary action, further providing continuous moisture and nutrients for seed germination and seedling growth. Natural gas exchange was permitted by the upper opening of the containers, which relied on air diffusion to maintain oxygen availability in the rhizosphere. A total of 6 seeds were placed on each medium. Dandelions were collected and ground. The calculation of indicators and selection of samples were determined based on the actual number of germinated dandelions (Figure A1).

The Se concentration in dandelion was determined by an inductively coupled plasma mass spectrometer (ICP-MS; DIONEX AQUION, Thermo Scientific™). Prior to analysis, the dried sample was subjected to microwave-assisted acid digestion by concentrated HNO_3_ to mineralize the organic matrix and release Se into solution. The digestion program was optimized to ensure complete decomposition of the plant material while minimizing volatile Se losses. After digestion, the solution was cooled, diluted to a final volume with ultrapure water, and introduced into the ICP-MS system for analysis.

The glucose-equivalent total sugars were determined by the phenol-sulfuric acid method [31,32]. Small molecule metabolites were detected by LC-MS to investigate the effect of PKCH on dandelion polysaccharide levels. All details are shown in Appendix A. To minimize the risk of tissue damage and subsequent leakage of intracellular contents, the rinsed roots were gently blotted dry and then immersed in 40 mL of DI water for 6 h, then filtered through a 0.45 μm membrane. A procedural blank (water without roots) was included to distinguish active exudation from passive leakage artifacts. The solution was concentrated to 1 mL and filtered again through a 0.22 μm membrane. Organic acids (oxalic acid and acetic acid) were quantified by HPLC at 214 nm by external calibration curves (1–200 μg/mL and 5–500 μg/mL, respectively; R^2^ > 0.99), with identification based on retention times compared to authentic standards. The limits of detection were 0.15 μg/mL for oxalic acid and 0.45 μg/mL for acetic acid. The mobile phase consisted of 0.5% (NH_4_)_2_HPO_4_-H_3_PO_4_ (pH = 2.5).

### 4.6. Statistical Analysis

All experiments were performed in triplicate (technical replicates), with six seeds sown in each culture medium (biological replicates). The experiment was designed as a one-factor comparison of different substrate types under identical nutrient solution conditions. The samples for crop growth indicator assessment were selected based on the actual germination rate. Statistical analysis was performed by one-way ANOVA followed by Tukey’s HSD post hoc test for multiple comparisons. Differences were considered statistically significant at *p* < 0.05. Experimental results are expressed as mean ± standard deviation.

## Data Availability

Data are contained within the article. All data are available upon request.

## Appendix A

Dried dandelion powder (100 mg) was transferred into a 2 mL centrifuge tube. Subsequently, aqueous methanol (0.6 mL, 70% *v*/*v*) was added as the extraction solvent. The mixture was vigorously vortexed and then centrifuged at 10,000 rpm for 10 min at room temperature to separate the supernatant from the solid residue. To ensure complete precipitation of polysaccharides and to enhance the stability of the target analytes, the supernatant was collected and incubated at 4 °C overnight and for 12 h. Finally, the supernatant was filtered through a 0.22 μm membrane filter prior to transfer into an autosampler vial for subsequent LC-MS analysis. The chromatographic separation was performed on a Waters UPLC HSS T3 C18 column (2.1 mm × 100 mm, 1.8 μm). The mobile phase consisted of solvent A (ultrapure water with 0.04% acetic acid) and solvent B (acetonitrile with 0.04% acetic acid). The gradient elution program was as follows: 0 min, 5% B; 0–10.00 min, increased to 95% B and held for 1 min; 11.00–11.10 min, returned to 5% B, followed by equilibration until 14 min. The flow rate was 0.35 mL·min^−1^, the column temperature was maintained at 40 °C, and the injection volume was 4 μL.

**Table A1:** TGA values of prepared hydrogels.

Material	Thermal Degradation Step	T _onset_ (°C)	T _endset_ (°C)	Weight Loss (%)
PKH-1	I	30	173	18.69
	II	173	1000	55.55
PKH-2	I	30	173	13.31
	II	173	1000	53.63
PKH-3	I	30	173	16.44
	II	173	1000	53.22
PKH-4	I	30	173	14.35
	II	173	1000	56.64
PKH-5	I	30	173	14.64
	II	173	1000	55.23

**Table A2:** Surface area, pore volume and pore width of PKH nanoparticles. Samples were tested with nitrogen sorption at 273 K.

PKH	PKH-1	PKH-2	PKH-3	PKH-4	PKH-5
BET surface area (m^2^/g)	28.10	29.39	37.72	39.56	47.29
Langmuir Surface Area (m^2^/g)	26.86	36.98	39.07	46.87	59.77
Total pore volume (cm^3^/g) at p/p_0_ = 0.019	0.054	0.057	0.076	0.076	0.093
Adsorption pore Diameter (nm)	5.001	4.139	3.789	3.389	3.018

**Table A3:** Quantitative data logs.

Time (d)	Temperature (°C)	Humidity (%)	Light
1	22.5 ± 1.2	35 ± 5	natural sunlight
2	22.3 ± 1.2	36 ± 5	natural sunlight
3	22.0 ± 1.3	38 ± 5	natural sunlight
4	21.8 ± 1.3	37 ± 5	natural sunlight
5	21.5 ± 1.3	29 ± 5	natural sunlight
6	21.0 ± 1.5	30 ± 6	natural sunlight
7	20.8 ± 1.2	32 ± 6	natural sunlight
8	20.5 ± 1.5	31 ± 6	natural sunlight
9	21.0 ± 1.5	33 ± 6	natural sunlight
10	20.8 ± 1.5	34 ± 6	natural sunlight
11	20.5 ± 1.5	35 ± 7	natural sunlight
12	20.2 ± 1.5	36 ± 7	natural sunlight
13	20.0 ± 1.5	36 ± 7	natural sunlight
14	22.8 ± 1.5	34 ± 6	natural sunlight
15	21.5 ± 1.5	33 ± 5	natural sunlight
16	22.4 ± 1.5	32 ± 5	natural sunlight
17	21.8 ± 1.5	30 ± 6	natural sunlight
18	20.7 ± 1.5	33 ± 5	natural sunlight
19	20.5 ± 1.5	34 ± 5	natural sunlight
20	21.3 ± 1.5	36 ± 5	natural sunlight

**Table A4:** The quality parameters of dandelions.

Name	Solution	Fresh Weight (g)	Dry Weight (g)
Water	Water	5.58 ± 1.21	1.54 ± 0.09
Kelcogel	Water	4.75 ± 0.09	1.10 ± 0.06
PKH	Water	5.96 ± 0.34	1.69 ± 0.04
PKCH	Water	7.47 ± 0.46	1.84 ± 0.07
Water	SL	3.94 ± 0.68	1.02 ± 0.05
Kelcogel	SL	3.86 ± 0.77	0.78 ± 0.03
PKH	SL	5.19 ± 0.24	1.15 ± 0.10
PKCH	SL	7.45 ± 1.19	1.83 ± 0.08
Water	MS	10.63 ± 0.81	3.14 ± 0.47
Kelcogel	MS	7.56 ± 0.77	1.91 ± 0.14
PKH	MS	8.62 ± 0.85	2.70 ± 0.04
PKCH	MS	11.23 ± 0.85	3.43 ± 0.10

**Table A5:** Chemical characterization of SL.

pH	Electrical Conductivity µS·cm^−1^	TOC g kg^−1^	TN g kg^−1^
7.68	103.56 ± 2.33	12.32 ± 0.20	1.09 ± 0.27

**Table A6:** The physiological parameters of dandelions.

Name	Solution	Shoot Length (cm)	Root Length (cm)	Main Root Diameter (cm)
Water	Water	11.59 ± 1.71	12.27 ± 1.63	0.45 ± 0.23
Kelcogel	Water	10.41 ± 2.16	5.42 ± 0.61	0.74 ± 0.19
PKH	Water	13.03 ± 2.13	7.01 ± 0.51	0.76 ± 0.17
PKCH	Water	14.48 ± 1.58	8.32 ± 1.52	0.83 ± 0.17
Water	SL	5.45 ± 0.95	4.33 ± 1.83	0.47 ± 0.11
Kelcogel	SL	7.98 ± 1.60	5.35 ± 0.45	0.55 ± 0.12
PKH	SL	7.76 ± 2.08	5.36 ± 1.56	0.61 ± 0.04
PKCH	SL	9.01 ± 1.81	6.13 ± 1.53	0.71 ± 0.08
Water	MS	12.21 ± 2.33	13.83 ± 2.16	0.59 ± 0.06
Kelcogel	MS	11.71 ± 1.93	7.71 ± 1.46	0.76 ± 0.12
PKH	MS	15.54 ± 2.19	7.76 ± 0.86	0.81 ± 0.10
PKCH	MS	16.32 ± 1.66	8.15 ± 1.45	0.92 ± 0.09

**Table A7:** The physiological parameters of dandelions in long-term experiment.

Name	Solution	Shoot Length (cm)	Root Length (cm)	Main Root Diameter (cm)
Water	MS	15.87 ± 0.25	16.10 ± 0.56	0.68 ± 0.07
Kelcogel	MS	14.41 ± 0.76	8.23 ± 0.09	0.85 ± 0.03
PKH	MS	17.23 ± 0.44	8.89 ± 0.18	0.88 ± 0.05
PKCH	MS	18.36 ± 0.30	9.28 ± 0.21	0.94 ± 0.02

**Table A8:** The quality parameters of dandelions in long-term experiment.

Name	Solution	Fresh Weight (g)	Dry Weight (g)
Water	MS	14.46 ± 0.94	3.17 ± 0.38
Kelcogel	MS	9.39 ± 0.36	2.33 ± 0.28
PKH	MS	9.67 ± 0.37	3.63 ± 0.22
PKCH	MS	15.97 ± 0.58	3.58 ± 0.21

**Table A9:** Content of organic acids and polysaccharides.

Name	Oxalic Acid (mg/kg)	Acetic Acid (mg/kg)	Polysaccharides (%)
Water	6.56 ± 0.23	3.41 ± 0.19	64.20 ± 1.06
Kelcogel	6.92 ± 0.21	3.75 ± 0.18	66.31 ± 0.91
PKH	8.72 ± 0.20	4.13 ± 0.34	68.15 ± 0.89
PKCH	10.66 ± 0.66	4.33 ± 0.28	69.40 ± 0.38

**Table A10:** Technical economic analysis of the PKCH synthesis system.

Project	Parameter/Description	Value/USD	Remarks
CAPEX (Capital Expenditure)		2.1 M	
Reactor system	multi-stage continuous mixing tank (316L stainless steel)	850,000	Corrosion-resistant design, temperature control accuracy ± 1 °C
Purification module	Ultrafiltration + ion exchange integrated system	620,000	Membrane flux ≥ 50 L/(m^2^·h) The recovery rate (90%)
Drying and granulation equipment	Spray drying + fluidized bed granulation	430,000	The particle size (50–100 μm)
Automatic control system	DCS + online monitoring (pH/viscosity/selenium content)	200,000	Sensor calibration and redundancy design
OPEX (Operating Expenditure)		1.35 M/yr	
Material	pectin (Orange peel low degree, food grade)	480,000/yr	Unit price 12 USD/kg, purity > 85%
Kelcogel	Kelcogel + Se/chitosan	320,000/yr	Se load (0.8 wt%)
Energy consumption	Electricity + steam (0.8 MPa)	290,000/yr	Dry energy consumption accounts for 65%
Catalyst and reagent	Enzyme catalyst (pectinase + transsulfase)	150,000/yr	enzyme activity retention rate ≥ 80%/5 batches
Effluent disposal	COD removal (biological + membrane filtration)	110,000/yr	Reuse rate ≥ 70%
Income		3.8 M/yr	
Sales revenue of PKCH products	Unit price: USD 3800/t	3.8 M/yr	Based on the pricing of functional food additives market
Net proceeds (NPV)	Discount rate 10%, 10-year cycle	6.2 M	IRR = 24.7%, investment payback period 3.8 years

**Table A11:** Life cycle assessment of the PKCH synthesis system.

Index	Value	Compared with Traditional Process (Baseline)
Greenhouse gas emission	2.1 kg CO_2_-eq/kg PKCH	decrease 38% (baseline: 3.4 kg)
Water consumption	4.8 m^3^/t PKCH	reduce 52% (baseline: 10 m3)
Energy intensity	18.7 MJ/kg PKCH	decrease 29% to (baseline: 26.3 MJ)

**Table A12:** Technical economic analysis of the PKCH soilless cultivation system.

Project	Parameter/Description	Value/USD	Remarks
CAPEX (Capital Expenditure)		1.8 M	
PKCH hydroponic substrate module	Customized porous gel scaffold (containing selenium-controlled release)	750,000	Life span of 5 years, support root air permeability and nutrient release
Environment control system	Light + temperature and humidity + CO_2_ regulation integration	520,000	Optimized LED spectrum, energy efficiency > 85%
Nutrient solution circulation purification system	Membrane filtration + UV sterilization + ion balance	380,000	Water recycling rate ≥ 95%
Automatic monitoring platform	Sensor network +AI growth model	150,000	Real-time monitoring of pH, EC and selenium content
OPEX (Capital Expenditure)		680,000/yr	
PKCH supplement cost	Annual consumption 12 t (unit price 25,000 USD/t)	300,000/yr	The gel degradation rate (8%/year)
Energy cost	Power rate (0.15 USD/kWh)	180,000/yr	Lighting (55%), environmental control (30%)
Labor and maintenance	Technicians (3) + equipment maintenance	120,000/yr	Includes sensor calibration and membrane component replacement
Seeds and consumables	Dandelion seedlings + nutrient solution additives	80,000/yr	Medicinal varieties, germination rate ≥ 90%
Income		2.5 M/yr	
Dandelion product sales revenue	Unit price USD 50,000/t (pharmaceutical grade)	2.5 M/yr	The market demand is stable and used for anti-inflammatory preparation production
Net proceeds (NPV)	Discount rate 8%, 10-year cycle	4.1 M	IRR = 28.3%, investment payback period 3.1 years

(Benchmark: annual production of 50 tons of dandelion).

**Table A13:** Life cycle assessment of PKCH soilless cultivation system.

Index	Value	Compared with Traditional Process (Baseline)
Greenhouse gas emission	1.2 kg CO_2_-eq/kg Dandelion	decrease 60% (baseline: 3.0 kg)
Water consumption	0.5 m^3^/t Dandelion	reduce 90% (baseline: 5 m^3^)
Soil occupancy	0 (soilless cultivation)	decrease 100% (baseline: 10 m^2^/t)
Utilization of selenium resources	92%	The utilization rate of traditional fertilization method < 40%
